# Midline vs Peripherally Inserted Central Catheter for Outpatient Parenteral Antimicrobial Therapy

**DOI:** 10.1001/jamainternmed.2024.5984

**Published:** 2024-11-11

**Authors:** David Paje, Emily Walzl, Megan Heath, Elizabeth McLaughlin, Jennifer K. Horowitz, Caitlin Tatarcuk, Lakshmi Swaminathan, Scott Kaatz, Anurag N. Malani, Valerie M. Vaughn, Steven J. Bernstein, Scott A. Flanders, Vineet Chopra

**Affiliations:** 1 Division of Hospital Medicine, Department of Internal Medicine, Michigan Medicine, Ann Arbor; 2 The Hospital Medicine Safety Consortium Coordinating Center, Ann Arbor, Michigan; 3 Medicine Service, VA Ann Arbor Healthcare System, Ann Arbor, Michigan; 4 Section of Hospital Medicine, Trinity Health Michigan, Ann Arbor; 5 Division of Hospital Medicine, Henry Ford Health, Detroit, Michigan; 6 Section of Infectious Diseases, Trinity Health Michigan, Ann Arbor; 7 Division of General Internal Medicine, Department of Internal Medicine, University of Utah, Salt Lake City; 8 Division of General Medicine, Department of Internal Medicine, Michigan Medicine, Ann Arbor; 9 Center for Clinical Management Research, VA Ann Arbor Healthcare System, Ann Arbor, Michigan; 10 Department of Medicine, University of Colorado Anschutz Medical Campus, Aurora

## Abstract

**Importance:**

Little is known about the safety of midline catheters vs peripherally inserted central catheters (PICCs) for outpatient parenteral antimicrobial therapy (OPAT).

**Objective:**

To compare outcomes from midline catheters vs PICCs for OPAT.

**Design, Setting, and Participants:**

This retrospective cohort study included patients who received antimicrobial therapy through a midline catheter or PICC between January 2017 and November 2023 across 69 Michigan hospitals. Because peripherally compatible OPAT was the indication of interest, vancomycin therapy was excluded. Data were analyzed from April to June 2024.

**Exposures:**

Insertion of a midline catheter or PICC for OPAT following hospitalization.

**Main Outcomes and Measures:**

The primary outcome was major device complications (ie, catheter-related bloodstream infection or catheter-related venous thromboembolism). Secondary outcomes included minor device complications (eg, catheter dislodgement, occlusion, tip migration, infiltration, superficial thrombophlebitis, or exit site concerns) and device failure, defined as catheter removal following device complication. Cox proportional hazards regression models were fit to device type and outcomes, adjusting for patient and device confounders and device dwell.

**Results:**

Of 2824 included patients, 1487 (53.5%) were male, and the median (IQR) age was 66.8 (55.9-77.1) years. Of 2824 devices placed for OPAT, 1999 (70.8%) were midline catheters and 825 (29.2%) were PICCs. The median (IQR) dwell time was 12 (8-17) days for midline catheters and 19 (12-27) days for PICCs (*P* < .001). A major device complication occurred in 44 patients (1.6%) overall, including 16 (0.8%) with midline catheters and 28 (3.4%) with PICCs (*P* < .001). OPAT delivered via midline catheters was associated with a lower risk of major complications vs PICCs (adjusted hazard ratio [aHR], 0.46; 95% CI, 0.23-0.91). Risks of minor complications and device failure were similar across device types (minor complications: 206 of 1999 [10.3%] vs 114 of 825 [13.8%]; aHR, 1.07; 95% CI, 0.83-1.38; device failure: 191 of 1999 [9.6%] vs 100 of 825 [12.1%]; aHR, 1.26; 95% CI, 0.96-1.65). For device dwell of 14 or fewer days, midline catheters were associated with a lower risk of major complications (12 of 1324 [0.9%] vs 16 of 304 [5.3%]; aHR, 0.29; 95% CI, 0.12-0.68) and similar risk of failure (151 of 1324 [11.4%] vs 52 of 304 [17.1%]; aHR, 0.79; 95% CI, 0.56-1.12) vs PICCs. For dwell longer than 14 days, no significant difference in rates of major complications (4 of 675 [0.6%] vs 12 of 521 [2.3%]; aHR, 0.42; 95% CI, 0.13-1.40) or device failure (40 of 675 [5.9%] vs 48 of 521 [9.2%]; aHR, 1.02; 95% CI, 0.64-1.61) were observed.

**Conclusions and Relevance:**

In this study, midline catheters appeared to be safe alternatives to PICCs for OPAT, particularly if infusions were planned for 14 or fewer days.

## Key Points

**Question:**

Are midline catheters associated with similar or lower risk of major device complications compared with peripherally inserted central catheters (PICCs) in patients receiving outpatient parenteral antimicrobial therapy (OPAT)?

**Findings:**

In this cohort study that included 2824 vascular access devices placed for OPAT, the risk of major device complications was lower with midline catheters compared with PICCs, particularly for devices that dwelled for 14 or fewer days.

**Meaning:**

These findings suggest that midline catheters are safe alternatives to PICCs for OPAT, especially for treatment durations of 14 or fewer days.

## Teaser

This cohort study compares outcomes from midline catheters vs peripherally inserted central catheters for outpatient parenteral antimicrobial therapy.

## Introduction

Outpatient parenteral antimicrobial therapy (OPAT) is often indicated for patients with serious infections requiring treatment beyond their hospital stay.^1,2,3^ Peripherally inserted central catheters (PICCs) are commonly placed to administer OPAT, owing to their ease of use and reliability in the outpatient setting.^4,5,6^ In recent years, midline catheters are increasingly being used instead of PICCs for OPAT.^7,8^

Similar to PICCs, midline catheters are inserted in the peripheral veins of the upper extremities. However, midline catheters are shorter in length and terminate in the peripheral veins rather than the cavoatrial junction.^9^ Thus, unlike PICCs, midline catheters are not appropriate for medications classified as vesicants or irritants whose delivery should be limited to central venous catheters.^10,11^ Current practice guidelines recommend midline catheters over PICCs for peripherally compatible infusates if the anticipated duration of therapy is 14 or fewer days; however, evidence supporting this recommendation is limited.^3,9,10^

Few studies have examined outcomes of midline catheters compared with PICCs specifically for the indication of OPAT.^8,12,13^ To bridge this gap, we used data from the Michigan Hospital Medicine Safety (HMS) Consortium to assess outcomes from midline catheter vs PICC use in hospitalized patients discharged with OPAT. We hypothesized that midline catheters would have similar or lower rates of major device complications compared with PICCs for peripherally compatible therapies.

## Methods

### Study Setting

The HMS Consortium is a statewide multihospital collaborative quality initiative sponsored by Blue Cross Blue Shield of Michigan and Blue Care Network that aims to improve the quality of care for hospitalized medical patients by reducing patient safety events. The consortium design and study protocols have been previously described.^14,15^ The institutional review board at the University of Michigan classified this study as not regulated (HUM00078730), and informed consent was waived. This study followed the Strengthening the Reporting of Observational Studies in Epidemiology (STROBE) reporting guideline.

Of the 92 nonfederal, noncritical access hospitals in Michigan, 69 (75%) participate in the HMS Consortium and share patient-level data on PICC and midline catheter placements. These include rural hospitals, small hospitals (fewer than 250 beds), large community hospitals (375 or more beds), and academic medical centers. At each hospital, an HMS-trained and consortium-supported abstractor collects data from electronic health records on a sample of midline catheters and PICCs placed in medical and critically ill patients. At each hospital, patient sampling and data collection occurred using a standard protocol and data collection tool.^14,16^ Data were captured from the time of device insertion to device removal, death, or 30 days following placement, whichever occurred first. Follow-up was performed through manual review of medical records, including clinical documentation and results of imaging and laboratory/microbiological tests, and through patient telephone interviews. Patients were sampled consecutively from general medical and intensive care units over a 14-day cycle, with the first 6 patients meeting inclusion criteria enrolled. As the focus of HMS Consortium is adult hospitalized medical patients, patients that were (1) younger than 18 years, (2) pregnant, (3) admitted for palliative care, (4) admitted to a nonmedical service (eg, surgery), or (5) admitted under observation status were excluded. The coordinating center at University of Michigan performs annual audits at each participating hospital to assure data quality and integrity.

### Patients

**Figure 1.**
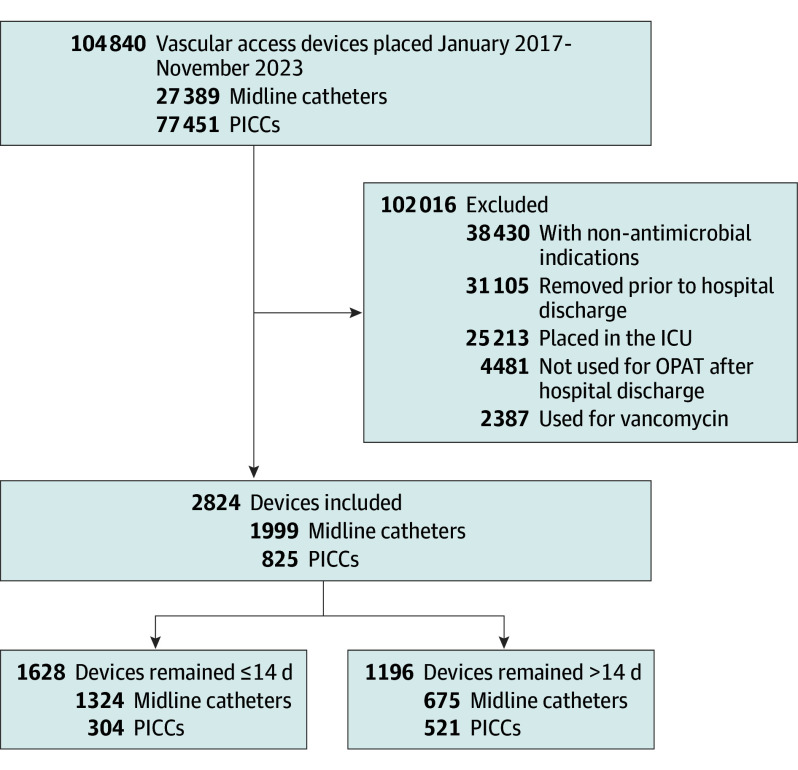
Flow Diagram for Cohort Selection ICU indicates intensive care unit; OPAT, outpatient parenteral antimicrobial therapy; PICC, peripherally inserted central catheter.

To mirror contemporary practice, we used data abstracted from the medical records of patients who received a midline catheter or PICC placed between January 2017 and November 2023 while admitted to a general medical unit at a participating hospital. To define an OPAT cohort, we selected patients whose documented indication for midline catheter or PICC placement was parenteral antimicrobial therapy, began treatment during hospitalization, and continued such therapy beyond hospital discharge (Figure 1). This included patients who received their device on the day of or the day prior to hospital discharge. To ensure homogeneity of our cohort and identify a pure OPAT population, we excluded patients who received a device placed in an intensive care unit setting. We also excluded patients who received vancomycin, an antibiotic that is both a vesicant and an irritant to the vascular endothelium and not recommended for prolonged courses of treatment via a midline catheter in existing guidelines.^10,17,18,19^

### Covariates

Patient characteristics, including demographic data, detailed clinical history, medications, level of care, laboratory test results (including estimated glomerular filtration rate), and discharge location, were abstracted from the electronic health records at the time of device insertion. Race data were self-reported. Comorbidities were quantified using the Charlson Comorbidity Index.^20^ Clinician characteristics (eg, operator who performed the device insertion and number of insertion attempts) and device characteristics (eg, number of lumens, catheter gauge, power vs nonpower, and device material) were collected from the device insertion note. Hospital characteristics, including number of beds and teaching status, were obtained from HMS Consortium and publicly reported hospital information.^21,22,23^

### Outcomes

The primary outcome was the occurrence of a major device complication, ie, documented catheter-related bloodstream infection (CRBSI) or catheter-related venous thromboembolism (CR-VTE). Among patients with PICCs, CRBSI events included central line–associated bloodstream infection (CLABSI) in accordance with the US Centers for Disease Control and Prevention/National Healthcare Safety Network criteria of laboratory-confirmed bloodstream infection (BSI) with a PICC in place for at least 48 hours without another identified source of BSI or physician documentation of catheter line sepsis, catheter-related bacteremia/fungemia, or PICC removal due to suspected CLABSI.^24^ Among those with midline catheters, CRBSI was defined as laboratory-confirmed BSI with a midline catheter in place and no other identified source of BSI or physician documentation of catheter line sepsis, catheter-related bacteremia/fungemia, or midline catheter removal due to suspected CRBSI. CR-VTE included symptomatic image-confirmed upper-extremity deep vein thrombosis and symptomatic image-confirmed pulmonary embolism.

The secondary outcomes were minor complications (eg, catheter dislodgement, occlusion, tip migration, infiltration, superficial thrombophlebitis, and exit site concerns [including leaking, discharge, or infection]) and device failure, which was defined as removal of the device because of any complication. We chose this composite secondary outcome because (1) premature removal is a meaningful end point for both patients and clinicians and (2) device complications are often competing events where the occurrence of a complication (eg, occlusion) may influence the probability of another (eg, phlebitis). Thus, using premature device removal as a distinct end point better encapsulates clinical impact of these events. We used validated, published definitions to capture and record all major and minor complications.^21,25^

### Statistical Analysis

Descriptive statistics were used to summarize patient and device data. Patients that received midline catheters vs PICCs were compared across patient, clinician, and device characteristics, using χ^2^ or Wilcoxon tests for categorical and continuous variables, respectively. The association between outcomes and type of device (midline catheter vs PICC) were assessed using a Cox proportional hazards model, accounting for catheter dwell time. Models were adjusted for patient age, sex, Charlson Comorbidity Index score, history of venous thromboembolism, history of CLABSI, active malignant neoplasm, receipt of anticoagulants, presence of another central vein catheter, number of catheter lumens, and catheter size. Random-effects models were used to account for hospital-level variation. As current practice guidelines endorse midline catheter use for 14 or fewer days and there are very limited data to suggest that midline catheters are safe beyond this period, we stratified our assessment of the association by dwell time, using 14 days as the cutoff.^3,26^ We also performed sensitivity analyses limited to patients whose discharge location was home (instead of a postacute care facility).

Results were expressed as adjusted hazard ratios (aHRs) with 95% CIs. All tests were 2-tailed, and *P* < .05 was considered statistically significant. All analyses were performed in SAS version 9.4 (SAS Institute).

## Results

Of 2824 included patients, 1487 (53.5%) were male, and the median (IQR) age was 66.8 (55.9-77.1) years. Of 2824 devices placed for OPAT, 1999 (70.8%) were midline catheters and 825 (29.2%) were PICCs. The median (IQR) Charlson Comorbidity Index score was 3 (1-5). Most patients (1964 [69.5%]) were discharged from hospital to home. However, 768 (27.2%) were transferred to a postacute care facility, including 525 of 1999 (26.3%) with midline catheters and 243 of 825 (29.5%) with PICCs (*P* = .11) (Table 1).

**Table 1.** Patient, Clinician, and Device Characteristics of Outpatient Parenteral Antimicrobial Therapy (OPAT) by Device Type

Characteristic	No. (%)		
	Total (N = 2824)	Midline catheter (n = 1999)	PICC (n = 825)
**Patient characteristics**			
Age, y			
18-49	466 (16.5)	349 (17.5)	117 (14.2)
50-69	1175 (41.6)	796 (39.8)	379 (45.9)
≥70	1183 (41.9)	854 (42.7)	329 (39.9)
Sex			
Female	1294 (46.5)	960 (48.8)	334 (41.1)
Male	1487 (53.5)	1009 (51.2)	478 (58.9)
Self-reported race			
Black	344 (12.2)	240 (12.0)	104 (12.6)
White	2328 (82.4)	1641 (82.1)	687 (83.3)
Other or unknown race^a^	152 (5.4)	118 (5.9)	34 (4.1)
BMI^b^			
Underweight (<18.5)	87 (3.1)	64 (3.2)	23 (2.8)
Normal (18.5-24.9)	649 (23.0)	460 (23.0)	189 (23.1)
Overweight (25-29.9)	727 (25.8)	505 (25.3)	222 (27.2)
Obese (≥30)	1305 (46.3)	922 (46.1)	383 (46.9)
Unknown	48 (1.7)	48 (2.4)	0
Charlson Comorbidity Index score			
0-1	846 (30.0)	613 (30.7)	233 (28.2)
2-3	893 (31.6)	629 (31.5)	264 (32.0)
>3	1085 (38.4)	757 (37.9)	328 (39.8)
Length of hospital stay, median (IQR), d	6.0 (4.0-8.0)	5.0 (4.0-8.0)	6.0 (4.0-9.0)
Time in hospital prior to placement, median (IQR), d	5.0 (3.0-7.0)	5.0 (3.0-7.0)	5.0 (3.0-7.0)
Time in hospital after placement, median (IQR), d	1.0 (0-1.0)	0 (0-1.0)	1.0 (0-1.0)
Time of OPAT, median (IQR), d	12.5 (8.0-20.0)	11.0 (7.0-17.0)	18.0 (11.0-26.0)
Advanced CKD (eGFR <45 mL/min/1.73 m^2^)	331 (11.7)	226 (11.3)	105 (12.7)
Hemodialysis	21 (0.7)	15 (0.8)	6 (0.7)
Active malignant neoplasm	42 (1.5)	8 (0.4)	34 (4.1)
Receiving chemotherapy	10 (0.4)	7 (0.4)	3 (0.4)
Liver disease	59 (2.1)	50 (2.5)	9 (1.1)
History of CLABSI	30 (1.1)	21 (1.1)	9 (1.1)
History of VTE	385 (13.6)	259 (13.0)	126 (15.3)
Receiving anticoagulant	1762 (62.4)	1220 (61.0)	542 (65.7)
Presence of CVC	33 (1.2)	25 (1.3)	8 (1.0)
PICC or CVC in prior 3 mo	227 (8.0)	133 (6.7)	94 (11.4)
Indications for device placement			
Antibiotics	2824 (100)	1999 (100)	825 (100)
Difficult access	96 (3.4)	65 (3.3)	31 (3.8)
Chemotherapy	1 (<0.1)	1 (0.1)	0
Multiple fluids	2 (0.1)	0	2 (0.2)
TPN	6 (0.2)	0	6 (0.7)
Level of care at device placement			
Emergency department	6 (0.2)	3 (0.2)	3 (0.4)
Inpatient medical floor	2814 (99.6)	1992 (99.6)	822 (99.6)
Intensive care unit	0	0	0
Other or unknown	4 (0.1)	4 (0.2)	0
Discharge location			
Home	1964 (69.5)	1413 (70.7)	551 (66.8)
Postacute care facility	768 (27.2)	525 (26.3)	243 (29.5)
Other or unknown	92 (3.3)	61 (3.1)	31 (3.8)
**Clinician characteristics**			
Health care professional inserting device			
Vascular access nurse	2376 (89.1)	1767 (96.0)	609 (73.8)
Interventional radiologist	192 (7.2)	45 (2.4)	147 (17.8)
Advanced practice professional (IR)	29 (1.1)	29 (1.6)	0
Other or unknown	69 (2.6)	0	69 (8.4)
Infectious disease specialist consulted	2441 (86.4)	1728 (86.4)	713 (86.4)
Teaching hospital	2667 (96.1)	1919 (97.8)	748 (91.9)
Hospital size			
<250 Beds	979 (35.3)	634 (32.3)	345 (42.4)
250-374 Beds	780 (28.1)	559 (28.5)	221 (27.1)
≥375 Beds	1017 (36.6)	769 (39.2)	248 (30.5)
Placement attempts			
1	2248 (79.6)	1587 (79.4)	661 (80.1)
≥2	214 (7.6)	162 (8.1)	52 (6.3)
Unknown	362 (12.8)	250 (12.5)	112 (13.6)
Device characteristics			
Single lumens	2635 (98.0)	1854 (98.9)	781 (95.7)
Catheter size or thickness			
<5F Catheter	2496 (94.4)	1759 (96.7)	737 (89.3)
≥5F Catheter	148 (5.6)	60 (3.3)	88 (10.7)
Power capable	2344 (83.0)	1578 (78.9)	766 (92.8)
Dwell time, d			
≤5 d	217 (7.7)	187 (9.4)	30 (3.6)
6-14 d	1411 (50.0)	1137 (56.9)	274 (33.2)
≥15 d	1196 (42.4)	675 (33.8)	521 (63.2)

Abbreviations: BMI, body mass index; CKD, chronic kidney disease; CLABSI, central line–associated bloodstream infection; CVC, central venous catheter; eGFR, estimated glomerular filtration rate; IR, interventional radiology; PICC, peripherally inserted central catheter; TPN, total parenteral nutrition; VTE, venous thromboembolism.

^a^ The other race category included American Indian or Alaska Native, Arab and Chaldean Ancestries, Asian, Native Hawaiian or Other Pacific Islander, and others.

^b^ Calculated as weight in kilograms divided by height in meters squared.

Most devices were placed on the day of or day prior to hospital discharge. Device insertion was successful on the first attempt for most midline catheters (1587 of 1999 [79.4%]) and PICCs (661 of 825 [80.1%]; *P* = .22). Vascular access nurses performed almost all midline catheter insertions (1767 [96.0%]) and most PICC insertions (609 [73.8%]); interventional radiologists placed 147 PICCs (17.8%) in this study. Infectious disease specialists evaluated 2441 of 2824 patients (86.4%) regardless of device type. Compared with PICCs, midline catheters were more likely to be single-lumen catheters (1854 [98.9%] vs 781 [95.7%]; *P* < .001) and catheters smaller than 5F in size (1759 [96.7%] vs 737 [89.3%]; *P* < .001) but were less likely to be capable of power injection (1578 [78.9%] vs 766 [92.8%]; *P* < .001). The median (IQR) catheter dwell time was 12 (8-17) days for midline catheters and 19 (12-27) days for PICCs (*P* < .001).

### Device-Related Complications With OPAT

A major device complication occurred in 44 patients (1.6%) in the overall cohort; 16 of 1999 patients with midline catheters (0.8%) and 28 of 825 those with PICCs (3.4%; *P* < .001) (Table 2). Overall, CRBSI was reported in 20 patients (0.7%), whereas image-confirmed CR-VTE was documented in 26 patients (0.9%). Patients with midline catheters experienced fewer CRBSI (5 [0.3%] vs 15 [1.8%]; *P* = .001) and CR-VTE events (11 [0.6%] vs 15 [1.8%]; *P* = .001) compared with those with PICCs.

**Table 2.** Frequency of Device Complications in Patients Receiving Outpatient Parenteral Antimicrobial Therapy by Device Type

Outcome	No. (%)			*P* value
	Total (N = 2824)	Midline catheter (n = 1999)	PICC (n = 825)	
Any major complication	44 (1.6)	16 (0.8)	28 (3.4)	<.001
Catheter-related BSI	20 (0.7)	5 (0.23)	15 (1.8)	<.001
Catheter-related VTE	26 (0.9)	11 (0.6)	15 (1.8)	.001
Upper-extremity DVT	22 (0.8)	11 (0.6)	11 (1.3)	.03
Pulmonary embolism	4 (0.1)	0	4 (0.5)	.002
Any minor complication	320 (11.3)	206 (10.3)	114 (13.8)	.007
Catheter dislodgement	120 (4.2)	75 (3.8)	45 (5.5)	.04
Catheter occlusion	82 (2.9)	45 (2.3)	37 (4.5)	.001
Catheter tip migration	58 (2.1)	22 (1.1)	36 (4.4)	<.001
Infiltration	9 (0.3)	9 (0.5)	0	NA
Superficial thrombosis	18 (0.6)	16 (0.8)	2 (0.2)	.09
Exit site concerns	81 (2.9)	72 (3.6)	9 (1.1)	<.001
Any major or minor complication	352 (12.5)	215 (10.8)	137 (16.6)	<.001
Device failure	291 (10.3)	191 (9.6)	100 (12.1)	.04

Abbreviations: BSI, bloodstream infection; DVT, deep vein thrombosis; NA, not applicable; PICC, peripherally inserted central catheter; VTE, venous thromboembolism.

Minor complications occurred in 320 patients (11.3%), including 206 (10.3%) with midline catheters and 114 (13.8%) with PICCs (*P* = .007). The most common minor complication in the overall cohort was catheter dislodgement (120 of 2824 [4.2%]), followed by catheter occlusion (82 of 2824 [2.9%]), and exit site concerns (81 of 2824 [2.9%]). For midline catheters, the most common minor complication was catheter dislodgement (75 [3.8%]), followed by exit site concerns (72 [3.6%]) and occlusion (45 [2.3%]). For PICCs, catheter dislodgement (45 [5.5%]) was the most common minor complication, followed by occlusion (37 [4.5%]) and tip migration (36 [4.4%]). Device failure occurred in 291 patients (10.3%), including 191 (9.6%) with midline catheters and 100 (12.1%) with PICCs (*P* = .04).

**Figure 2.**
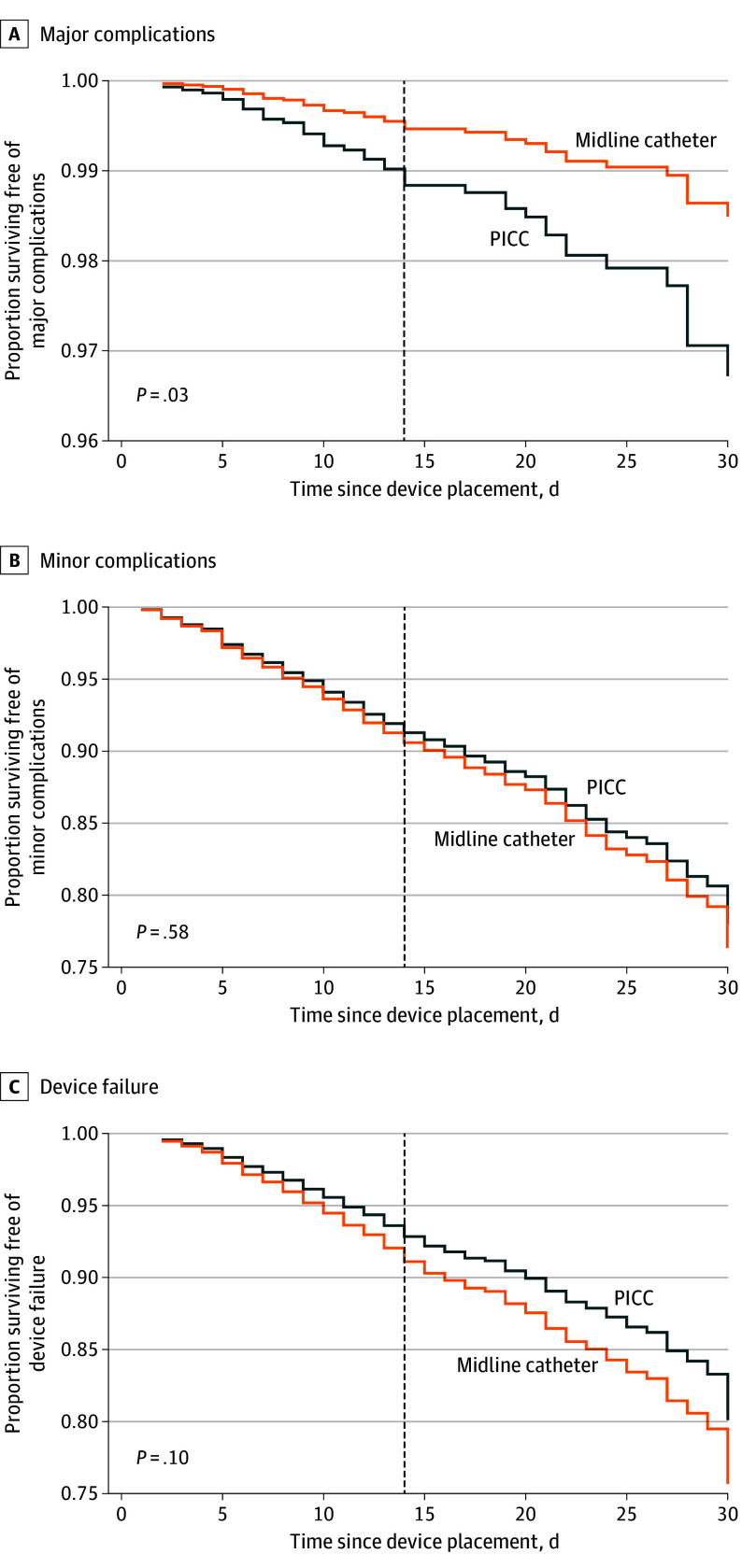
Complications in Patients Receiving Outpatient Parenteral Antimicrobial Therapy by Device Type PICC indicates peripherally inserted central catheter.

After accounting for dwell time and covariates, midline catheters placed for OPAT had a lower risk of major complications compared with PICCs (aHR, 0.46; 95% CI, 0.23-0.91). However, no significant difference in CRBSI (aHR, 0.37; 95% CI, 0.11-1.19) or CR-VTE (aHR, 0.64; 95% CI, 0.26-1.59) were observed. When examining secondary outcomes, midline catheters were similar to PICCs with respect to minor complications (aHR, 1.07; 95% CI, 0.83-1.38) and device failure (aHR, 1.26; 95% CI, 0.96-1.65) (Table 3; Figure 2). In sensitivity analyses limited to patients whose discharge location was home, compared with PICCs, results were robust with regard to midline catheters’ lower risk of major complications (aHR, 0.35; 95% CI, 0.14-0.90) and similar risk of minor complications (aHR, 1.18; 95% CI, 0.87-1.61). However, midline catheters were more often associated with device failure compared with PICCs among patients discharged to home (aHR, 1.46; 95% CI, 1.04-2.06) (eTable 1 in Supplement 1).

**Table 3.** Incidence Density and Hazards of Device Complications in Outpatient Parenteral Antimicrobial Therapy by Device Type

Outcome	No. (No. per 1000 catheter-days)			Adjusted hazard ratio (95% CI)^a^	*P* value
	Total (N = 2824)	Midline catheter (n = 1999)	PICC (n = 825)		
Any major complication	44 (1.03)	16 (0.59)	28 (1.79)	0.46 (0.23-0.91)	.03
Catheter-related BSI	20 (0.47)	5 (0.18)	15 (0.96)	0.37 (0.11-1.19)	.10
Catheter-related VTE	26 (0.61)	11 (0.41)	15 (0.96)	0.64 (0.26-1.59)	.34
Upper-extremity DVT	22 (0.52)	11 (0.41)	11 (0.7)	0.64 (0.26-1.60)	.34
Pulmonary embolism	4 (0.1)	0	4 (0.26)	NA	NA
Any minor complication	320 (7.49)	206 (7.62)	114 (7.28)	1.07 (0.83-1.38)	.58
Catheter dislodgement	120 (2.81)	75 (2.77)	45 (2.87)	1.00 (0.67-1.51)	.99
Catheter occlusion	82 (1.92)	45 (1.66)	37 (2.36)	0.94 (0.53-1.67)	.84
Catheter tip migration	58 (1.36)	22 (0.81)	36 (2.3)	0.30 (0.16-0.55)	<.001
Infiltration	9 (0.21)	9 (0.33)	0	NA	NA
Superficial thrombosis	18 (0.42)	16 (0.59)	2 (0.13)	4.25 (0.84-21.55)	.08
Exit site concerns	81 (1.9)	72 (2.66)	9 (0.57)	4.01 (1.94-8.31)	<.001
Any major or minor complication	352 (8.24)	215 (7.95)	137 (8.75)	0.96 (0.76-1.21)	.70
Device failure	291 (6.81)	191 (7.06)	100 (6.39)	1.26 (0.96-1.65)	.10

Abbreviations: BSI, bloodstream infection; DVT, deep vein thrombosis; NA, not applicable; PICC, peripherally inserted central catheter; VTE, venous thromboembolism.

^a^ Cox proportional hazards model adjusted for age, sex, Charlson Comorbidity Index score, history of VTE, history of central line–associated bloodstream infection, active malignant neoplasm, receipt of anticoagulants, presence of another central vein catheter, number of catheter lumens, and catheter size, with random effects for each hospital.

### Differences in Outcomes by Dwell Time

A larger proportion of midline catheters than of PICCs dwelled for 14 or fewer days (1324 [66.2%] vs 304 [36.8%]). In this shorter dwell duration group, midline catheters were associated with a lower risk of major complications vs PICCs (12 of 1324 [0.9%] vs 16 of 304 [5.3%]; aHR, 0.29; 95% CI, 0.12-0.68) (eTable 2 and eFigure 1 in Supplement 1). However, no significant difference in minor complications (156 of 1324 [11.8%] vs 44 of 304 [14.5%]; aHR, 0.88; 95% CI, 0.61-1.25) and device failure (151 of 1324 [11.4%] vs 52 of 304 [17.1%]; aHR, 0.79; 95% CI, 0.56-1.12) between midline catheters and PICCs were noted.

A smaller proportion of midline catheters than of PICCs were in place beyond 14 days (675 [33.8%] vs 521 [63.2%]). In this longer dwell group, no statistically significant difference in rates of major complications (4 of 675 [0.6%] vs 12 of 521 [2.3%]; aHR, 0.42; 95% CI, 0.13-1.40), minor complications (50 of 675 [7.4%] vs 70 of 521 [13.4%]; aHR, 0.73; 95% CI, 0.49-1.09), or device failure (40 of 675 [5.9%] vs 48 of 521 [9.2%]; aHR, 1.02; 95% CI, 0.64-1.61) were observed between those that received a midline catheter or a PICC (eTable 3 and eFigure 2 in Supplement 1).

## Discussion

In this large multihospital study, we found a lower risk of major complications in patients receiving OPAT through a midline catheter compared with a PICC. CRBSI was infrequent, and the incidence of CR-VTE was low among patients with midline catheters. Minor complications and device failure were similar among those that received midline catheters vs PICCs. The lower risk of major complications with midline catheters was more pronounced in devices that dwelled for 14 or fewer days. These findings have important implications in selecting a vascular access device for the millions of patients who receive OPAT today.

The low rates of CRBSI from midline catheters observed in our cohort has been reported in other studies.^13,27,28,29,30,31^ However, our findings related to the risk of midline catheter–related thrombosis differ from others.^28,29,32,33^ One systematic review that pooled results from single-site, retrospective studies reported a greater risk of thrombosis with midline catheters compared with PICCs.^34^ Prior analyses using data from multiple sites also suggested greater risk of thrombosis with midline catheters vs PICCs, particularly in patients requiring vascular access for short-term durations, and in patients who received vasopressors through midline catheters.^12,35^ However, a more recent meta-analysis found no difference in rates of catheter-related deep vein thrombosis or pulmonary embolism between device types.^31^ To our knowledge, our study is the first to examine this aspect in a cohort of patients transitioning from inpatient to outpatient settings and specifically in those receiving antimicrobials. The low rates of CR-VTE observed in this study may relate to surveillance bias (limited testing in outpatients) or to reduction in risk from differing patient cohorts or less frequent device use as may occur during hospitalization.

When stratified by dwell time, we found that patients with midline catheters in place for 14 or fewer days had significantly lower risk of major complications, particularly CR-VTE, vs PICCs. Further, during this short duration of midline catheter use, the risks of minor complications and device failure were similar to PICCs. For dwell exceeding 14 days, the likelihood of any major complication occurring with midline catheters was similar to those with PICCs. The rates of minor complications and device failure were also comparable in both devices beyond 14 days. Collectively, these findings support current practice that prefer midline catheters over PICCs for peripherally compatible infusates if the intended use is 14 or fewer days.^9,10^ However, these findings also suggest that midline catheters may also be considered for dwell times beyond 14 days. Future studies evaluating factors such as specific infusates, varying care practices, and device placement techniques or properties that support longer midline catheter dwell would be welcomed.^17,18,19,36^

Minor complications often necessitate device removal or replacement, interrupting OPAT and causing patient discomfort or inconvenience. Consistent with previous reports, we found minor complications to be common with both midline catheters and PICCs.^14,16,25^ The most common midline catheter complication was catheter dislodgement, which in many cases necessitated device removal. Catheter occlusion was also common with midline catheters and generally led to premature midline catheter removal, as little evidence examining the use of thrombolytics to declot midline catheters is available.^37^ In contrast, catheter occlusion of PICCs is often remediable with thrombolytics and may not necessitate device removal. Studies examining the safety and efficacy of the use of these agents in midline catheters appear necessary.

### Limitations and Strengths

Our study has limitations. First, we collected data from medical records of hospitalized patients; our findings are therefore limited to what was documented for clinical care and available for abstraction. Second, as with all observational studies, we cannot account for unknown confounders, and our results should be interpreted as hypothesis generating rather than causal. Third, we did not capture catheter-related complications that may have occurred after device removal or beyond 30 days of follow-up. This could result in underestimation of the risk of these events. Fourth, we did not have information on dosing and combination regimens of antimicrobial agents; larger studies examining effects of dosing frequency or complex therapies are needed to truly determine the safety of these 2 devices. While we excluded vancomycin, it is important to note that practice trends for treating Methicillin-resistant *Staphylococcus aureus* and other resistant gram-positive infections with OPAT are changing rapidly. Less toxic effective agents that do not require intensive therapeutic monitoring are increasingly preferred over vancomycin.^38,39,40,41,42,43,44^ Thus, we do not believe that exclusion of this agent represents a major limitation in relation to contemporary practice. Similarly, many patients prescribed OPAT may not require intravenous therapy. Future work should assess appropriateness and necessity of OPAT and whether oral options might be safer. Finally, while we did not detect differences in major complications and device failure rates between midline catheters and PICCs beyond 14 days, our study was not adequately powered for this analysis. Future, larger studies that focus on characteristics associated with longer-term device dwells appear necessary.

Despite these limitations, our study has many strengths. First, to our knowledge, this is one of the first and largest studies examining outcomes from OPAT in patients with midline catheters vs PICCs. As we used data from a large multi-institutional collaborative that included rural hospitals and large referral centers, our findings are likely highly generalizable to various practice settings. Second, we used a robust data collection strategy, which included trained abstractors at each hospital, standardized forms and data definitions, and routine audits of data quality at each site. Third, aside from capturing major morbid events, we also accounted for minor complications, such as catheter dislodgement and occlusion. In addition, we examined device failure—a meaningful end point for patients and clinicians. These outcomes help inform clinical care in novel and important ways.

## Conclusions

In conclusion, in this study, we found midline catheters to be safe alternatives to PICCs for patients who are prescribed OPAT after hospitalization, particularly if the intended duration is 14 or fewer days. These results strengthen recommendations from existing appropriateness criteria and guidelines for use of these devices. Studies that examine optimal devices and drug strategies for OPAT beyond 14 days would be welcomed.

## Notes

Supplement 1. **eFigure 1.** Complications in Patients Receiving OPAT ≤14 Days by Device Type **eFigure 2.** Complications in Patients Receiving OPAT >14 Days by Device Type **eTable 1.** Hazards of OPAT Device Complications Among Patients Discharged to Home by Device Type **eTable 2.** Hazards of OPAT Device Complications Among Devices With Dwell Time of ≤14 Days by Device Type **eTable 3.** Hazards of OPAT Device Complications Among Devices With Dwell Time of >14 Days by Device Type

Supplement 2. Data Sharing Statement
