# Supplementary material for: Midline vs Peripherally Inserted Central Catheter for Outpatient Parenteral Antimicrobial Therapy
Source: JAMA Intern Med. 2024 Nov 11;185(1):83–91. doi: 10.1001/jamainternmed.2024.5984 (PMC11555572; doi:10.1001/jamainternmed.2024.5984)
Supplement: Supplement 1. — eFigure 1. Complications in Patients Receiving OPAT ≤14 Days by Device Type eFigure 2. Complications in Patients Receiving OPAT >14 Days by Device Type eTable 1. Hazards of OPAT Device Complications Among Patients Discharged to Home by Device Type eTable 2. Hazards of OPAT Device Complications Among Devices With Dwell Time of ≤14 Days by Device Type eTable 3. Hazards of OPAT Device Complications Among Devices With Dwell Time of >14 Days by Device Type [file jamainternmed-e245984-s001.pdf]

Supplemental Online Content

Paje D, Walzl E, Heath M, et al. Midline vs peripherally inserted central catheter for outpatient parenteral antimicrobial therapy. *JAMA Intern Med*. Published online November 11, 2024. doi:10.1001/jamainternmed.2024.5984

eFigure 1. Complications in Patients Receiving OPAT ≤14 Days by Device Type

eFigure 2. Complications in Patients Receiving OPAT >14 Days by Device Type

eTable 1. Hazards of OPAT Device Complications Among Patients Discharged to Home by Device Type

eTable 2. Hazards of OPAT Device Complications Among Devices With Dwell Time of ≤14 Days by Device Type

eTable 3. Hazards of OPAT Device Complications Among Devices With Dwell Time of >14 Days by Device Type

This supplementary material has been provided by the authors to give readers additional information about their work.

© 2024 Paje D et al. *JAMA Internal Medicine*.

eFigure 1. Complications in Patients Receiving OPAT ≤14 Days by Device Type

A. Major Complications

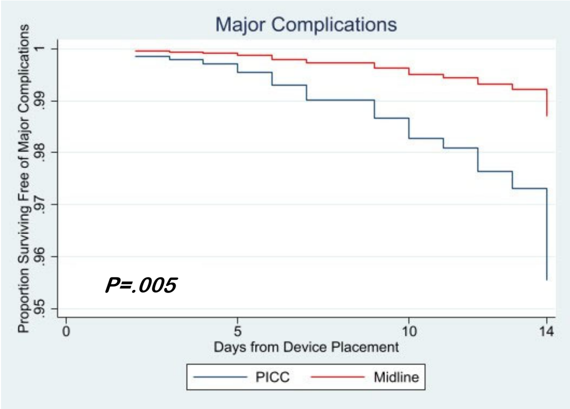

B. Minor Complications

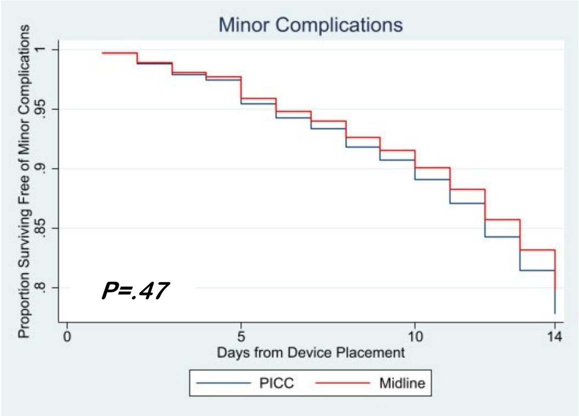

C. Device Failure

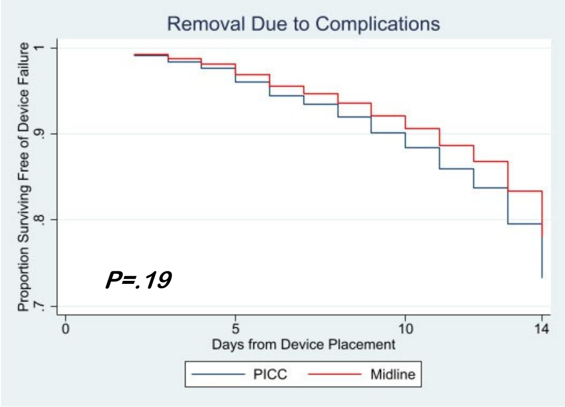

© 2024 Paje D et al. *JAMA Internal Medicine*.

eFigure 2. Complications in Patients Receiving OPAT >14 Days by Device Type

Major Complications

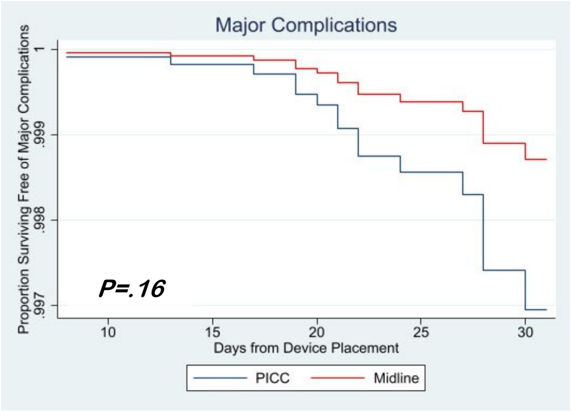

A. Minor Complications

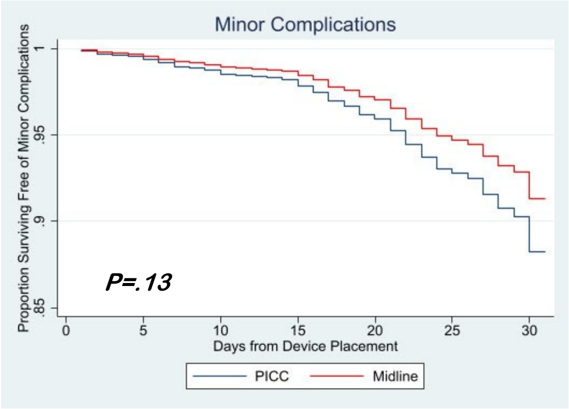

B. Device Failure

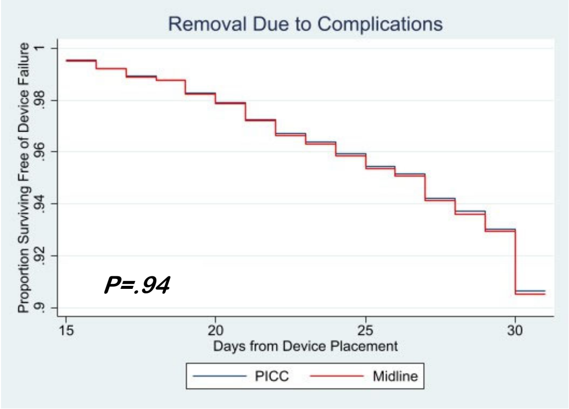

© 2024 Paje D et al. *JAMA Internal Medicine*.

eTable 1. Hazards of OPAT Device Complications Among Patients Discharged to Home by Device Type

Outcomes	Total (N = 1,964)	Midline (N = 1,413)	PICC (N = 551)	Adjusted Hazard Ratio*	95% Confidence Interval		*P-value*
	n (per 1,000 cd)	n (per 1,000 cd)	n (per 1,000 cd)		Lower Limit	Upper Limit	
**Any Major Complication**	24 (0.8)	9 (0.47)	15 (1.38)	0.35	0.14	0.90	.03
Catheter-related BSI	9 (0.3)	2 (0.1)	7 (0.65)	0.07	0.01	1.14	.06
Catheter-related VTE	16 (0.53)	7 (0.36)	9 (0.83)	0.51	0.16	1.60	.25
Upper extremity DVT	13 (0.43)	7 (0.36)	6 (0.55)	0.51	0.16	1.60	.25
Pulmonary embolism	3 (0.1)	0 (0)	3 (0.28)	NA	NA	NA	NA
**Any Minor Complication**	228 (7.57)	153 (7.94)	75 (6.92)	1.18	0.87	1.61	.29
Catheter dislodgement	77 (2.56)	51 (2.65)	26 (2.4)	1.22	0.72	2.09	.46
Catheter occlusion	63 (2.09)	37 (1.92)	26 (2.4)	1.06	0.54	2.11	.86
Catheter tip migration	46 (1.53)	20 (1.04)	26 (2.4)	0.39	0.20	0.74	.004
Infiltration	6 (0.2)	6 (0.31)	NA	NA	NA	NA	NA
Superficial thrombosis	14 (0.47)	14 (0.73)	0 (0)	NA	NA	NA	NA
Exit site concerns	64 (2.13)	56 (2.91)	8 (0.74)	3.10	1.43	6.73	.004
**Any Major or Minor Complication**	247 (8.2)	159 (8.25)	88 (8.12)	1.04	0.78	1.39	.79
**Device Failure**	201 (6.68)	141 (7.32)	60 (5.54)	1.46	1.04	2.06	<.001

Legend: OPAT=outpatient parenteral antimicrobial therapy; PICC=peripherally inserted central catheter; cd=catheter-days; BSI=bloodstream infection; VTE=venous thromboembolism; DVT=deep vein thrombosis; *Hazard Ratio=Cox Proportional Hazards model adjusted for age, sex, Charlson comorbidity score, history of VTE, history of central line-associated bloodstream infection, active malignancy, receipt of anticoagulants, presence of another central vein catheter, number of catheter lumens, and catheter size, with random effects for each hospital

© 2024 Paje D et al. *JAMA Internal Medicine*.

eTable 2. Hazards of OPAT Device Complications Among Devices With Dwell Time of ≤14 Days by Device Type

Outcomes	Total (N = 1,628)	Midline (N = 1,324)	PICC (N = 304)	Adjusted Hazard Ratio*	95% Confidence Interval		*P-value*
	n (per 1,000 cd)	n (per 1,000 cd)	n (per 1,000 cd)		Lower Limit	Upper Limit	
**Any Major Complication**	28 (1.83)	12 (0.98)	16 (5.22)	0.29	0.12	0.68	.005
Catheter-related BSI	10 (0.66)	3 (0.25)	7 (2.28)	0.18	0.03	1.10	.06
Catheter-related VTE	20 (1.31)	9 (0.74)	11 (3.59)	0.35	0.13	0.99	.048
Upper extremity DVT	18 (1.18)	9 (0.74)	9 (2.94)	0.36	0.13	1.00	.05
Pulmonary embolism	2 (0.13)	0 (0)	2 (0.65)	NA	NA	NA	NA
**Any Minor Complication**	200 (13.11)	156 (12.79)	44 (14.35)	0.876	0.61	1.25	.47
Catheter dislodgement	85 (5.57)	60 (4.92)	25 (8.15)	0.53	0.32	0.89	.02
Catheter occlusion	36 (2.36)	29 (2.38)	7 (2.28)	1.57	0.53	4.68	.42
Catheter tip migration	29 (1.9)	20 (1.64)	9 (2.94)	0.47	0.20	1.08	.08
Infiltration	7 (0.46)	7 (0.57)	NA	NA	NA	NA	NA
Superficial thrombosis	17 (1.11)	15 (1.23)	2 (0.65)	1.67	0.35	8.09	.52
Exit site concerns	60 (3.93)	55 (4.51)	5 (1.63)	2.93	1.13	7.61	.03
**Any Major or Minor Complication**	219 (14.35)	162 (13.28)	57 (18.59)	0.74	0.53	1.02	.06
**Device Failure**	203 (13.3)	151 (12.38)	52 (16.96)	0.79	0.56	1.12	.19

Legend: OPAT=outpatient parenteral antimicrobial therapy; PICC=peripherally inserted central catheter; cd=catheter-days; BSI=bloodstream infection; VTE=venous thromboembolism; DVT=deep vein thrombosis; *Hazard Ratio=Cox Proportional Hazards model adjusted for age, sex, Charlson comorbidity score, history of VTE, history of central line-associated bloodstream infection, active malignancy, receipt of anticoagulants, presence of another central vein catheter, number of catheter lumens, and catheter size, with random effects for each hospital

© 2024 Paje D et al. *JAMA Internal Medicine*.

eTable 3. Hazards of OPAT Device Complications Among Devices With Dwell Time of >14 Days by Device Type

Outcomes	Total (N = 1,196)	Midline (N = 675)	PICC (N = 521)	Adjusted Hazard Ratio*	95% Confidence Interval		*P-value*
	n (per 1,000 cd)	n (per 1,000 cd)	n (per 1,000 cd)		Lower Limit	Upper Limit	
**Any Major Complication**	16 (0.58)	4 (0.27)	12 (0.95)	0.42	0.13	1.40	.16
Catheter-related BSI	10 (0.36)	2 (0.13)	8 (0.64)	0.33	0.06	1.81	.20
Catheter-related VTE	6 (0.22)	2 (0.13)	4 (0.32)	0.92	0.13	6.60	.94
Upper extremity DVT	4 (0.15)	2 (0.13)	2 (0.16)	0.93	0.13	6.61	.94
Pulmonary embolism	2 (0.07)	0 (0)	2 (0.16)	NA	NA	NA	NA
**Any Minor Complication**	120 (4.37)	50 (3.37)	70 (5.56)	0.73	0.49	1.09	.13
Catheter dislodgement	35 (1.28)	15 (1.01)	20 (1.59)	1.00	0.49	2.04	.99
Catheter occlusion	46 (1.68)	16 (1.08)	30 (2.38)	0.62	0.28	1.38	.24
Catheter tip migration	29 (1.06)	2 (0.13)	27 (2.14)	0.04	0.01	0.29	.001
Infiltration	2 (0.07)	2 (0.13)	NA	NA	NA	NA	NA
Superficial thrombosis	1 (0.04)	1 (0.07)	0 (0)	NA	NA	NA	NA
Exit site concerns	21 (0.77)	17 (1.14)	4 (0.32)	2.76	0.89	8.61	.08
**Any Major or Minor Complication**	133 (4.85)	53 (3.57)	80 (6.35)	0.69	0.47	1.01	.05
**Device Failure**	88 (3.21)	40 (2.69)	48 (3.81)	1.02	0.64	1.61	.94

Legend: OPAT=outpatient parenteral antimicrobial therapy; PICC=peripherally inserted central catheter; cd=catheter-days; BSI=bloodstream infection; VTE=venous thromboembolism; DVT=deep vein thrombosis; *Hazard Ratio=Cox Proportional Hazards model adjusted for age, sex, Charlson comorbidity score, history of VTE, history of central line-associated bloodstream infection, active malignancy, receipt of anticoagulants, presence of another central vein catheter, number of catheter lumens, and catheter size, with random effects for each hospital

© 2024 Paje D et al. *JAMA Internal Medicine*.
